# ESTIMATING CARDIORESPIRATORY FITNESS USING A USUAL-PACED 400 M–LONG DISTANCE CORRIDOR WALK IN OLDER ADULTS

**DOI:** 10.1093/geroni/igad104.1256

**Published:** 2023-12-21

**Authors:** Reagan Moffit, Peggy Cawthon, Bret Goodpaster, Frederico Toledo, Daniel Forman, Barb Nicklas, Anne Newman, Nancy Glynn

**Affiliations:** University of Pittsburgh School of Public Health, Pittsburgh, Pennsylvania, United States; California Pacific Medical Center, Research Institute, San Francisco, California, United States; AdventHealth, Orlando, Florida, United States; University of Pittsburgh, School of Medicine, Pittsburgh, Pennsylvania, United States; University of Pittsburgh, Pittsburgh, Pennsylvania, United States; Wake Forest School of Medicine, Winston Salem, North Carolina, United States; University, Pittburgh, Pennsylvania, United States; University of Pittsburgh School of Public Health, Pittsburgh, Pennsylvania, United States

## Abstract

Cardiopulmonary exercise testing (CPET) is the gold standard to measure cardiorespiratory fitness (CRF), assessed as peak oxygen consumption (VO2peak), yet is not always feasible in older adults due to cost, safety, and participant burden. Usual-paced 400-m Long Distance Corridor Walk (LDCW), a measure of mobility, may be an alternative to traditional CPET. We examined whether usual-paced 400-m LDCW was associated with VO2peak among older adults using cross-sectional data in SOMMA. Participants (N=820, age = 76.2±4.9, 58% women, 86% White) completed a usual-paced 400-m LDCW (seconds) and a treadmill CPET using a modified symptom-limited protocol at baseline. VO2peak (mL/kg/min) was defined as the highest 20-second average O2 consumption achieved during CPET. Mean VO2peak was 20.2±4.8 mL/kg/min and mean usual-paced 400-m LDCW time was 388.8±69.7 seconds. Usual-paced 400-m LDCW time was moderately inversely correlated with VO2peak (r=-0.52, p<0.0001). Potential covariates considered for the step-wise linear regression included age, sex, body mass index (BMI), Borg Rating of Perceived Exertion (RPE, range 6-20) at end of usual-paced 400-m LDCW, min/week moderate-vigorous physical activity (MVPA, CHAMPS questionnaire), 4-m gait speed, and Short Physical Performance Battery (range 0-12). The final parsimonious model included usual-paced 400-m LDCW time, age, sex, BMI, RPE, and MVPA. Every 30-second higher usual-paced 400-m LDCW time was associated with a 1.98 mL/kg/min lower VO2peak (p < 0.001), explaining 30.5% of the 55.7% total variance in VO2peak. Usual-paced 400-m LDCW was a strong estimator of CRF (VO2peak) after adjustment, providing an inexpensive and safer alternative among older adults across ranges of physical function and fitness.

